# Supplementary material for: The miR-290 and miR-302 clusters are essential for reprogramming of fibroblasts to induced pluripotent stem cells
Source: bioRxiv. 2024 Sep 3:2024.09.02.610895. Preprint. [Version 1] doi: 10.1101/2024.09.02.610895 (PMC11398367; doi:10.1101/2024.09.02.610895)
Supplement: Supplement 2 [file NIHPP2024.09.02.610895v1-supplement-2.pdf]

bioRxiv preprint doi: https://doi.org/10.1101/2024.09.02.610895; this version posted September 3, 2024. The copyright holder for this preprint (which was not certified by peer review) is the author/funder, who has granted bioRxiv a license to display the preprint in perpetuity. It is made available under aCC-BY 4.0 International license.

Supplemental Figures

Supplemental Figure 1

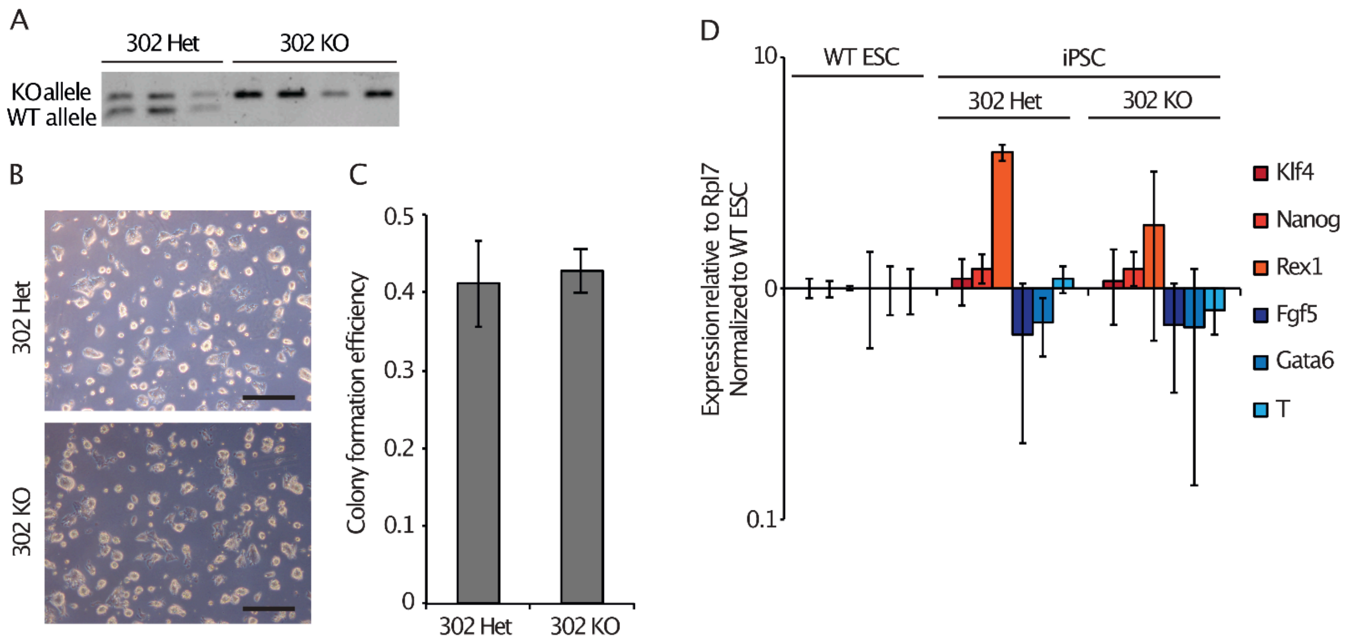

Supplemental Figure 1. (A) DNA genotyping results for the *mir-302~367* locus in *mir-302* Het and *mir-302* KO iPSC-like cell lines. (B) Representative brightfield images of reprogrammed and expanded *mir-302* Het and *mir-302* KO iPSC-like cells taken at 5x magnification (scale bar represents 500 um). (C) Colony formation efficiency (fraction of cells plated that form colonies) of *mir-302* Het and *mir-302* KO iPSC-like cells. (D) qRT-PCR analysis of naïve pluripotency markers (Klf4, Nanog, Rex1) and primed pluripotency markers (Fgf5, Gata6, T) in WT ESCs and *mir-302* Het and *mir-302* KO iPSC-like cells at P.12. Error bars represent SD of 3-4 biological replicates.

bioRxiv preprint doi: https://doi.org/10.1101/2024.09.02.610895; this version posted September 3, 2024. The copyright holder for this preprint (which was not certified by peer review) is the author/funder, who has granted bioRxiv a license to display the preprint in perpetuity. It is made available under aCC-BY 4.0 International license.

Supplemental Figure 2

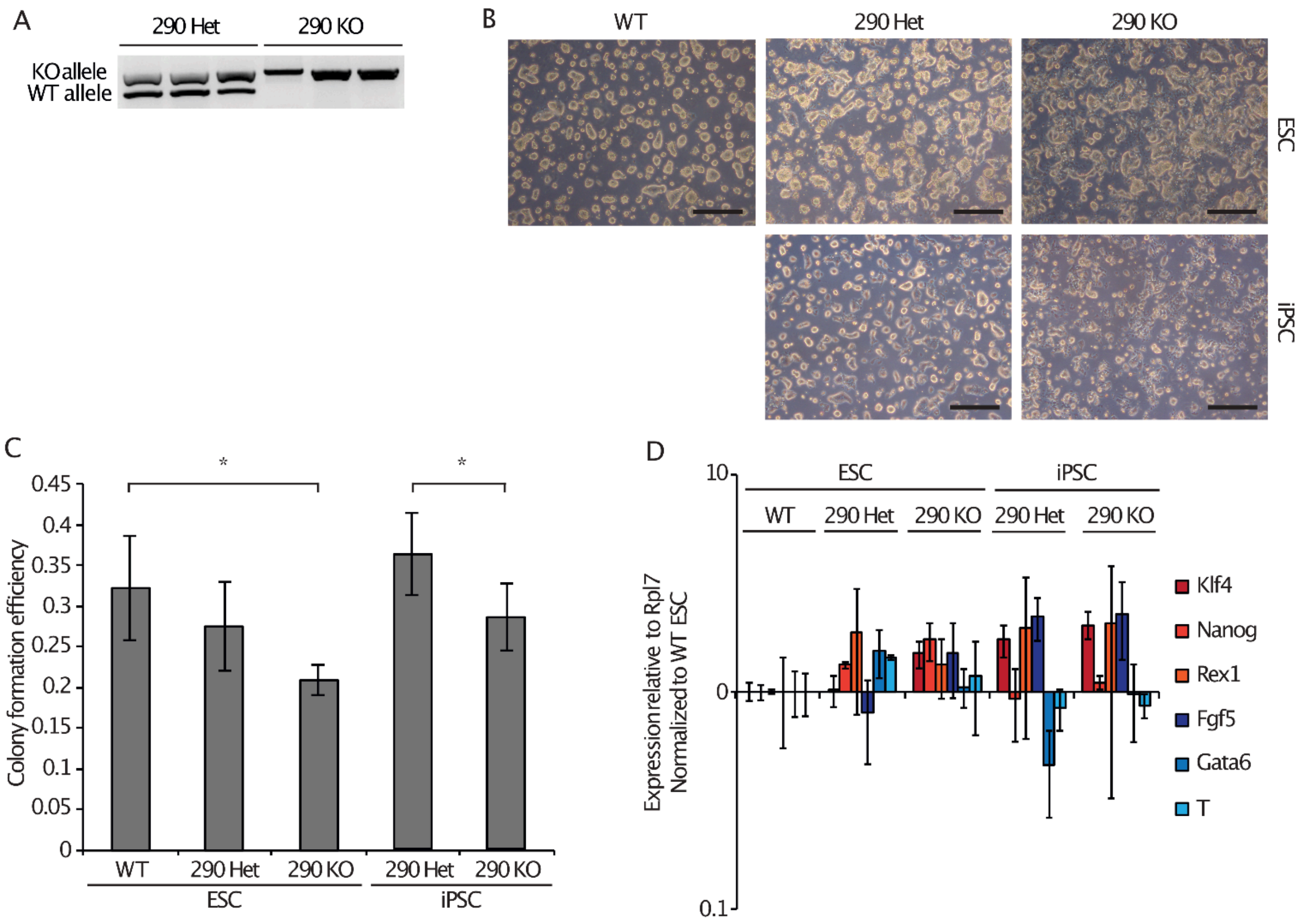

Supplemental Figure 2. (A) DNA genotyping results for the *mir-290~295* locus in the *mir-290* Het and *mir-290* KO iPSC-like cell lines. (B) Representative brightfield images of reprogrammed and expanded WT, *mir-290* Het, and *mir-290* KO ESCs and iPSCs taken at 5x magnification (scale bar represents 500 um). (C) Colony formation efficiency (fraction of cells plated that form colonies) of *mir-290* Het and *mir-290* KO ESCs and iPSC-like cells. (D) qRT-PCR analysis of naïve pluripotency markers (Klf4, Nanog, Rex1) and primed pluripotency markers (Fgf5, Gata6, T) in WT ESCs and *mir-290* Het and *mir-290* KO iPSC-like cells at P.12. Error bars represent SD of 3 biological replicates. (*) $P < 0.05$, two-sided t-test.

bioRxiv preprint doi: https://doi.org/10.1101/2024.09.02.610895; this version posted September 3, 2024. The copyright holder for this preprint (which was not certified by peer review) is the author/funder, who has granted bioRxiv a license to display the preprint in perpetuity. It is made available under aCC-BY 4.0 International license.

Supplemental Figure 3

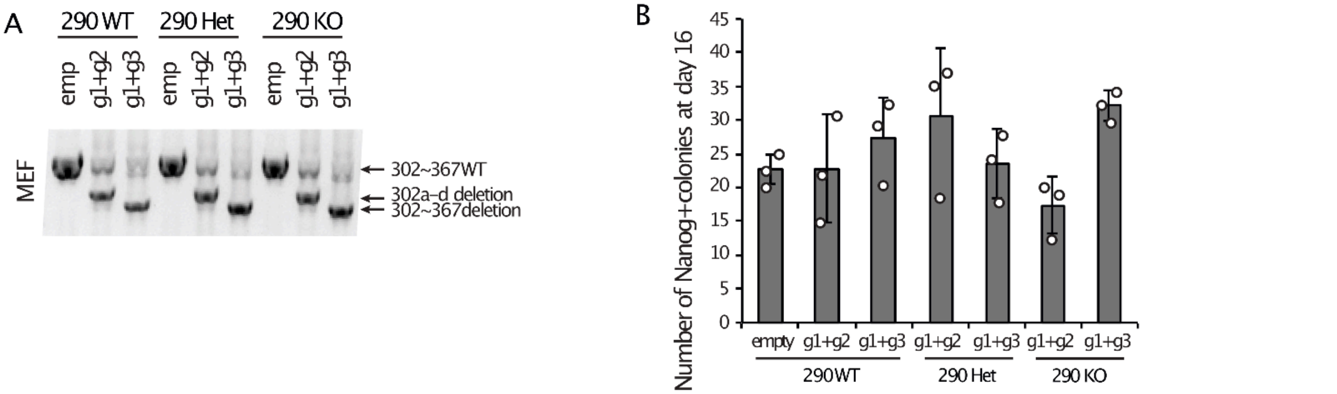

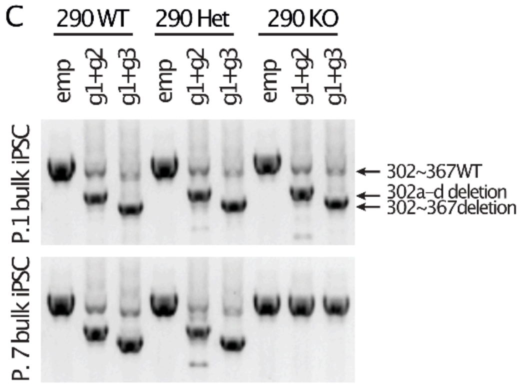

D

Number of P.7 iPSC clones with indicated 302+367genotypes

		302~367 +/+	302a–d −/−	302~367 −/−	302a–d +/−	302~367 +/−	Other	Total
290 +/+	302 empty	6	0	0	0	0	0	6
	302 g1+g2	10	2	0	0	0	0	12
	302 g1+g3	8	0	3	0	0	0	11
290 +/−	302 empty	6	0	0	0	0	0	6
	302 g1+g2	7	10	0	2	0	5	24
	302 g1+g3	7	0	6	0	7	3	23
290 −/−	302 empty	6	0	0	0	0	0	6
	290 KO g1+g2	36	0	0	0	0	0	36
	290 KO g1+g3	33	0	0	0	1	0	34

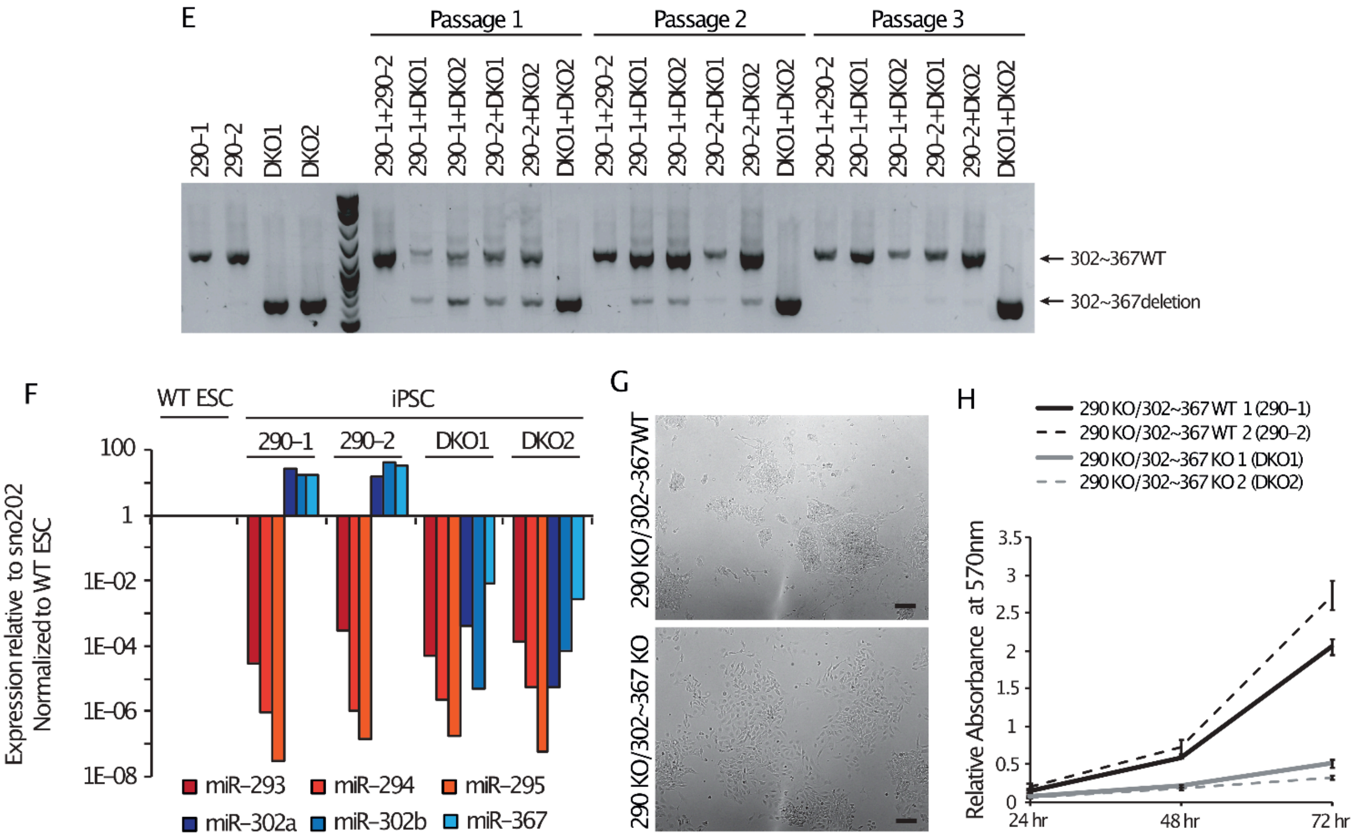

bioRxiv preprint doi: https://doi.org/10.1101/2024.09.02.610895; this version posted September 3, 2024. The copyright holder for this preprint (which was not certified by peer review) is the author/funder, who has granted bioRxiv a license to display the preprint in perpetuity. It is made available under aCC-BY 4.0 International license.

Supplemental Figure 3. (A) Genotyping results for the *mir-302~367* locus in the starting WT, *mir-290* Het, or *mir-290* KO MEF population. Arrows indicate PCR products representing WT *mir-302~367*, *mir-302a-d* deletion, and *mir-302~367* deletion. (B) Average number of Nanog+ colonies counted per well 16 days after OSK transduction. Cells electroporated with an empty CRISPR construct ("empty") were used as a control. Error bars represent SD of 3 biological replicates. Circles indicate individual data points. (C) Genotyping results for the *mir-302~367* locus in iPSCs harvested in bulk at P.1 and at P.7. (D) Number of individual iPSC clones of each indicated genotype picked at P.7. "Other" indicates non-WT PCR bands that are not consistent with either *mir-302a-d* or *mir-302~367* deletion. € Representative genotyping results over 3 consecutive passages when equal numbers of *mir-290-/-;mir-302~367+/+* (290-1, 290-2) and *miR-290-/-;mir-302~367-/-* (DKO1, DKO2) iPSCs were initially mixed. (F) Transcript levels relative to internal control and normalized to WT ESCs of mature *mir-290~295* and *mir-302~367* cluster miRNAs in WT ESCs, *mir-290-/-;mir-302~367+/+* (290-1, 290-2), and *miR-290-/-;mir-302~367-/-* (DKO1, DKO2) iPSC-like lines at P.12. (G) Representative images of *mir-290-/-;mir-302~367+/+* and *mir-290-/-;mir302~367-/-* iPSC-like lines taken at 10x magnification (scale bar represents 100 um). (H) Crystal violet absorbance levels in proliferation assay of two *mir-290-/-;mir-302~367+/+* (290-1, 290-2) and two *mir-290-/-;mir-302~367-/-* (DKO1, DKO2) iPSC lines. Absorbance is relative to blanked control. Error bars represent SD of 5 technical replicates.

bioRxiv preprint doi: https://doi.org/10.1101/2024.09.02.610895; this version posted September 3, 2024. The copyright holder for this preprint (which was not certified by peer review) is the author/funder, who has granted bioRxiv a license to display the preprint in perpetuity. It is made available under aCC-BY 4.0 International license.

Supplemental Figure 4

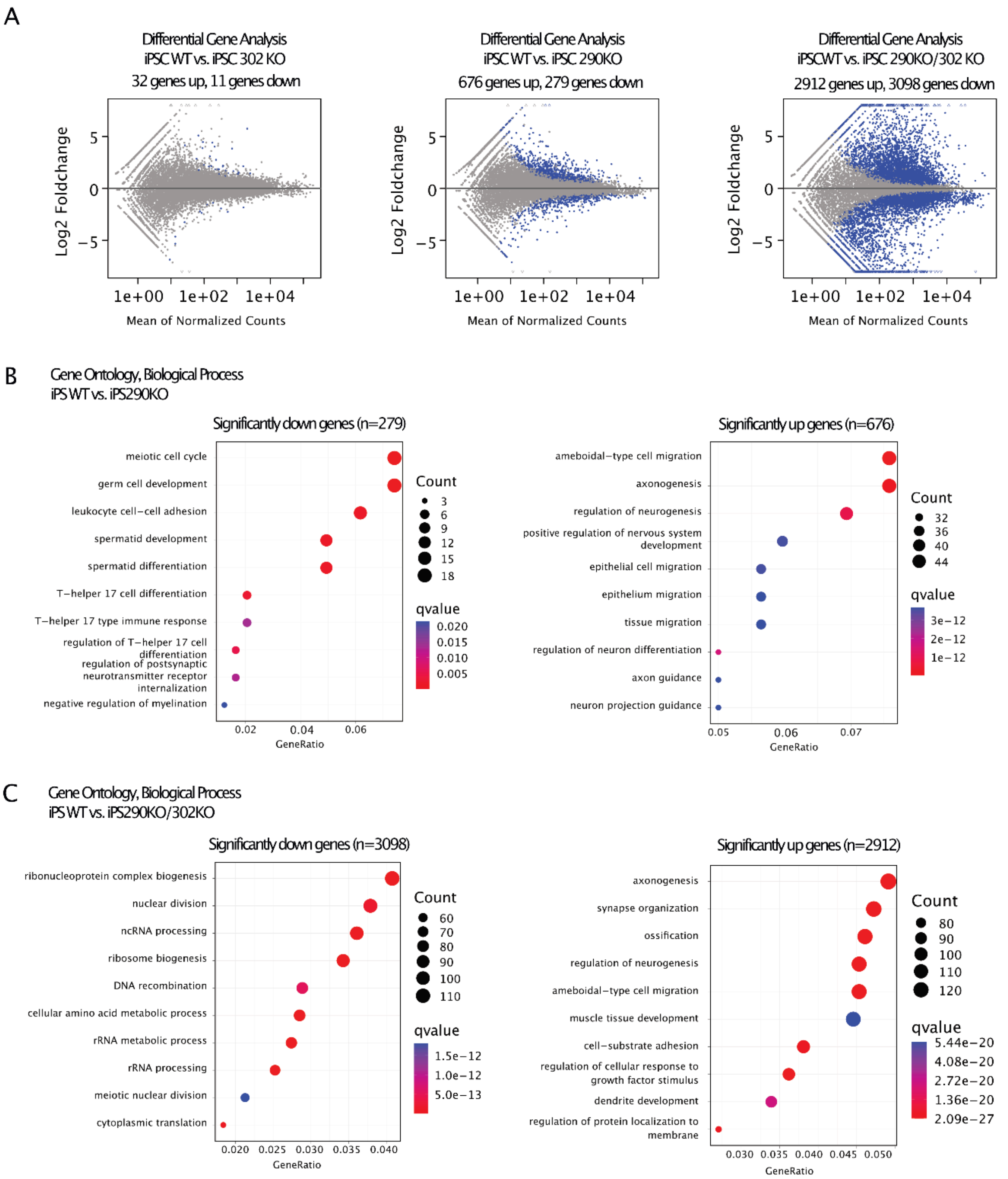

Supplemental Figure 4. (A) MA plots showing gene expression compared between WT iPSC and either 302KO iPSC-like cells, 290KO iPSC-like cells, or double KO iPSC-like cells. Significantly different genes highlighted in blue (Log2 Foldchange >1, p.value < 0.05 after correction using Benjamini-Hochberg method). (B) Top 10 gene ontology categories enriched using Clusterprofiler on differential genes identified using DESeq2 comparing WT vs. iPSC 290KO. (C) Same as (B) but comparing WT iPSCs vs double KO cells.

bioRxiv preprint doi: https://doi.org/10.1101/2024.09.02.610895; this version posted September 3, 2024. The copyright holder for this preprint (which was not certified by peer review) is the author/funder, who has granted bioRxiv a license to display the preprint in perpetuity. It is made available under aCC-BY 4.0 International license.

Supplemental Figure 5

A

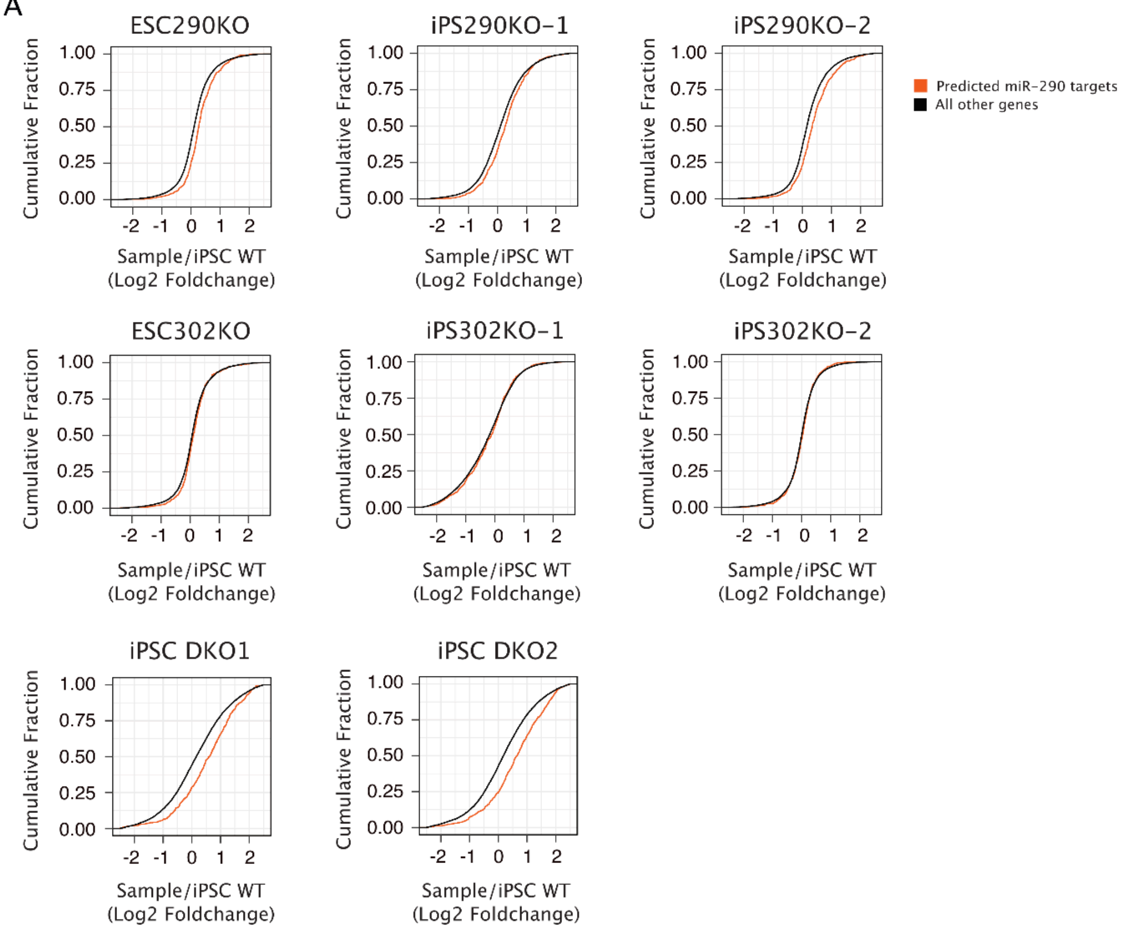

B

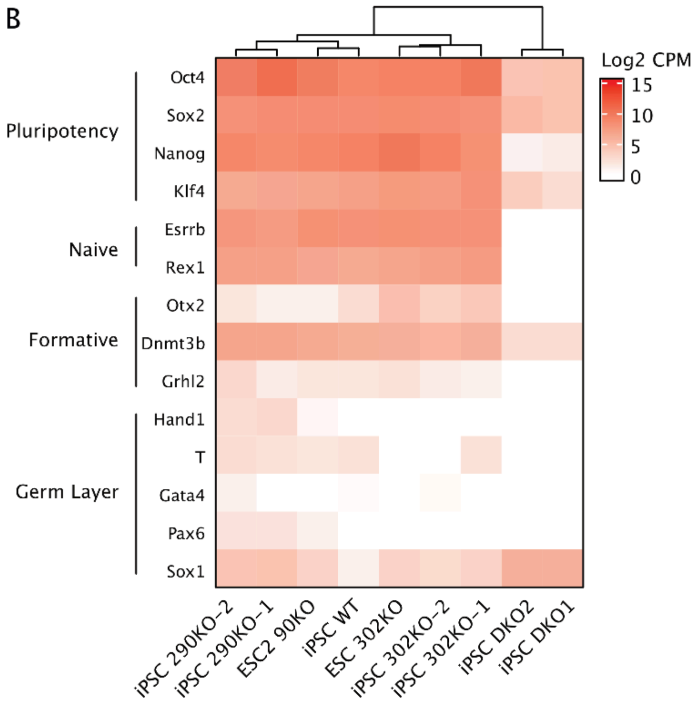

bioRxiv preprint doi: https://doi.org/10.1101/2024.09.02.610895; this version posted September 3, 2024. The copyright holder for this preprint (which was not certified by peer review) is the author/funder, who has granted bioRxiv a license to display the preprint in perpetuity. It is made available under aCC-BY 4.0 International license.

Supplemental Figure 5. (A) Plots comparing cumulative distribution functions of predicted miR-290/302 family targets (orange) to all other genes detected (black). Values on the X-axis are Log2 Foldchanges for each sample relative to iPSC WT cells. (B) A heatmap showing the absolute level of gene expression for select genes present in Figure 3H. Values represent Log2 counts per million (CPM). The plot is hierarchically clustered by sample.
